# Synthetic and Crystalline Amino Acids: Alternatives to Soybean Meal in Chicken-Meat Production

**DOI:** 10.3390/ani10040729

**Published:** 2020-04-22

**Authors:** Peter H. Selle, Juliano Cesar de Paula Dorigam, Andreas Lemme, Peter V. Chrystal, Sonia Y. Liu

**Affiliations:** 1 Poultry Research Foundation, The University of Sydney, Camden NSW2570, Australia; peter.selle@sydney.edu.au (P.H.S.); Peter_Chrystal@baiada.com.au (P.V.C.); 2 Evonik Nutrition and Care GmbH, 63457 Hanau-Wolfgang, Germanyandreas.lemme@evonik.com (A.L.); 3 Baiada Poultry Pty Limited, Pendle Hill NSW2145, Australia; 4 School of Life and Environmental Sciences, Faculty of Science, The University of Sydney, Camden NSW2570, Australia

**Keywords:** amino acids, broiler chickens, crude protein, digestive dynamics, soybean meal

## Abstract

**Simple Summary:**

There is a distinct possibility that synthetic and crystalline, or non-bound, amino acids will partially replace soybean meal in diets for broiler chickens and reduce the dependency of the chicken-meat industry on soybean meal as its principal source of protein. The genesis of this partial replacement will be the successful development of reduced-crude protein diets. A reduced-crude protein diet contains less soybean meal, and therefore less crude protein, but an increased array of essential and even non-essential non-bound amino acids so that requirements are met. There are, however, several challenges to be overcome if reduced-crude protein diets are to be successfully developed and adopted.

**Abstract:**

This review explores the premise that non-bound (synthetic and crystalline) amino acids are alternatives to soybean meal, the dominant source of protein, in diets for broiler chickens. Non-bound essential and non-essential amino acids can partially replace soybean meal so that requirements are still met but dietary crude protein levels are reduced. This review considers the production of non-bound amino acids, soybeans, and soybean meal and discusses the concept of reduced-crude protein diets. There is a focus on specific amino acids, including glycine, serine, threonine, and branched-chain amino acids, because they may be pivotal to the successful development of reduced-crude protein diets. Presently, moderate dietary crude protein reductions of approximately 30 g/kg are feasible, but more radical reductions compromise broiler performance. In theory, an ‘ideal’ amino acid profile would prevent this, but this is not necessarily the case in practice. The dependence of the chicken-meat industry on soybean meal will be halved if crude protein reductions in the order of 50 g/kg are attained without compromising the growth performance of broiler chickens. In this event, synthetic and crystalline, or non-bound, amino acids will become viable alternatives to soybean meal in chicken-meat production.

## 1. Introduction

Chicken-meat is the most rapidly growing source of protein for human consumption [1], which is environmentally advantageous as chicken-meat production generates less ‘greenhouse gases’ or carbon dioxide (CO_2_) equivalents than alternative meat protein sources. The production of 1 kg of chicken meat generates 1.1 kg CO_2_ equivalents, which is considerably less than that of pork (3.8 kg CO_2_ equivalents) or beef (14.8 kg CO_2_ equivalents) production [2]. Moreover, it has been predicted that chicken-meat production will contribute 7.1% of greenhouse gas emissions compared to 29.8% and 63.1% for pork and beef, respectively [2]. However, diets for broiler chickens may contain more than 200 g/kg protein, the majority of which is derived from soybean meal; consequently, the chicken-meat industry has a huge demand for soybean meal. The production of 1 kg of chicken meat will require an input in the order of 560 g soybean meal given a conservative 250 g/kg dietary inclusion of soybean meal and a 2.25:1 conversion of feed into carcass weight. Importantly, this demand can be diminished by inclusions of non-bound (synthetic and crystalline) amino acids in broiler diets at the expense of soybean meal via the successful development of reduced-crude protein (CP) diets [3]. Therefore, synthetic and crystalline amino acids are indeed alternatives to soybean meal in chicken-meat production and the purpose of this review is to explore the potential, and the challenges, of substituting non-bound amino acids for soybean meal in reduced-CP diets for broiler chickens. Such an exploration is justified because reduced-CP diets have the potential to halve the demand for soybean meal by the chicken-meat industry.

## 2. Amino Acid Production Processes

The inclusion of supplemental or non-bound amino acids in diets for poultry and livestock is an effective means to reduce the amount of resources, including land, feed, water, and energy, to generate more sustainable and efficient food production for an increasing world population. Additionally, it has been shown through life cycle assessments that amino acid additions to broiler diets have the potential to reduce greenhouse gas emissions in various regions, including Europe, and North and South America, compared to non-supplemented diets [4]. The industrial application of amino acids for animal feed has a long history. The production of *d,l*-methionine by chemical synthesis commenced in the late 1950s; whereas, the production of *l*-lysine by fermentation commenced during the 1960s and other amino acids, such as *l*-threonine and *l*-tryptophan, were introduced in the late 1980s [5]. The development of the market for amino acids over the past 40 years can be attributed to the cost-effective production and isolation of amino acid products [6]. Presently, amino acids are produced by three different methods: Extraction from protein hydrolysates, chemical synthesis, and microbial processes involving enzymatic synthesis and fermentation [7]. Each of these biotechnological processes have their economic and ecological advantages. 

### 2.1. Extraction from Protein Hydrolysates

The extraction from protein hydrolysates is not suitable for large-scale industrial production but is relevant for specific amino acids, including *l*-cysteine, *l*-leucine, and *l*-tyrosine [6,8]. Different extraction processes can be developed based on the different chemical affinity and pH of the amino acids for separation [9]. For instance, *l*-cysteine may be obtained from keratin, which is found in feathers, and can be extracted simply by using activated charcoal and concentrated hydrochloric acid [8]. Other amino acids, including *l*-leucine, *l*-alanine, and *l*-serine, can be produced from waste material derived from animals [7]. The main advantage of this method relies on the use of industrial by-products and wastes [10], which otherwise would be incinerated, contributing to increased greenhouse gases concentrations. Alternatively, this can also be considered the main bottleneck of the method, because it is highly dependent on the availability of natural protein-rich resources, making this process less competitive commercially [7].

### 2.2. Chemical Synthesis 

Chemical synthesis has been the classical pathway to produce racemic mixtures, such as *d,l*-methionine, that can be obtained by Strecker synthesis, which was initially proposed in 1850 [7]. The conversion of an aldehyde or ketone and amine or ammonia to α-amino acids can be achieved by means of an acid catalyst, a cyanide source, and water. The main disadvantage of chemical synthesis is its incapacity to selectively produce *d* or *l* forms of amino acids [11]. Methionine is produced on an industrial scale via the Bucherer–Bergs reaction, which is a variant of Strecker synthesis [7]. In this method, ketones or aldehydes are reacted with ammonium carbonate and sodium cyanide to produce hydantoins, which then undergo alkaline hydrolysis to racemic amino acid salt mixtures. Finally, *d,l*-methionine is crystallized following neutralization with sulphuric acid and carbon dioxide [10]. The industrial production of *d,l*-methionine uses conventional petrochemical raw materials, such as methyl mercaptan, acrolein, hydrocyanic acid, and ammonia [6]. Methionine is the first limiting amino acid for optimal growth in poultry offered maize-soybean based diets, so it is of particular importance. Indeed, it has been estimated that 1 kg of synthetic *d,l*-methionine supplies an equal quantity of digestible methionine as 178 kg of soybean meal [12]. The racemic *d,l*-methionine is widely accepted because chickens are able to convert the *d*-form to the biologically active *l*-form through oxidase and transaminase reactions. Therefore, the racemic *d,l*-methionine can be used without compromised efficiency [13]. There are currently different sources of methionine on the market, such as DL-Methionine 99% and the so-called 2-hydroxy-4-methylthio butanoic acid (HMTBa) also known as methionine hydroxy analog (MHA), which is found in liquid form (DL-MHA-FA 88%) and in powder (DL-MHA-Ca 84%). However, there is some evidence that HMTBa/HMTBa-Ca have a lower bioefficacy than DL-methionine on an equimolar/isosulfurous basis [14]. For other amino acids, such as lysine or threonine, there is no comparable enzyme system for converting the *d*-form into the active *l*-form; therefore, it is necessary to produce the *l*-form in high purity [6,12]. 

### 2.3. Enzymatic Process

The enzymatic process is based on the action of an enzyme or a combination of enzymes to catalyse the production of the desired amino acids. Several enzymes have been used, such as hydrolytic enzymes, ammonia lyases, and dehydrogenases [14]. Most of these enzymes are obtained from microorganisms, such as *Escherichia coli*, *Aspergillus oryzae*, and *Pseudomonas* sp. The main advantage of the enzymatic method is that it can produce pure *d*- and *l*-amino acids in high concentrations with a very low formation of by-products [8]. The classical example is the enzymatic conversion of *d,l*-methionine after acetylation to N-acetyl *d,l*-methionine, in which only the *l*-isomer is enzymatically converted by *l*-amino acylase from *Aspergillus oryzae* to obtain *l*-methionine [6,15,16]. The main drawback of this process is the high cost of the enzymes and their limited stability [8]. Therefore, this method is not preferable to produce *l*-amino acids at an industrial scale and it is used only to produce some amino acids, such as *l*-aspartic acid and *l*-alanine [17]. There is also the production of *l*-methionine via O-succinyl homoserine produced by fermentation followed by enzymatic reaction with methyl mercaptan [18], which is the same petrochemical used in the production of *d,l*-methionine. Therefore, *l*-methionine production is not entirely enzymatic in comparison to other sources.

### 2.4. Fermentation Process

The majority of processes for industrial amino acid production are based on the fermentation of microorganisms engineered for industrial application to convert carbon from carbohydrates and nitrogen from ammonia into specific amino acids. The fermentation process takes place in an agitated tank under sterile conditions. The culture medium contains a suitable carbon source, such as sugar cane syrup, as well as the required sources of nitrogen, sulfur, phosphorus, and some trace elements. A culture of the production strain is added to the fermentation tank and cultivated under mono-septic conditions with control of the temperature, pH, and aeration. For instance, the production of *l*-glutamic acid uses the bacterium strain *Corynebacterium glutamicum* and it is purified and obtained by crystallization in the recovery section of the fermentation plant [6]. The advances in fermentation technology have permitted the industrial-scale production of *l*-lysine, which also uses the same bacterium strain. In addition to the classic product form “lysine hydrochloride”, other forms, such as lysine sulphate and liquid lysine, have also become established, with differentiation regarding the application and production technology [6]. The post-fermentation process for *l*-lysine sulphate differs from that of *l*-lysine HCl [19,20], with its production being highly attractive for both ecological (with less liquid and solid waste) and economic reasons. This source is composed of *l*-lysine sulphate and fermentation by-products containing other amino acids, phosphorus, and energy, which are not present in the L-lysine HCl product [21]. *Corynebacterium glutamicum* is predominantly used in the synthesis of the branched-chain amino acids, *l*-isoleucine, *l*-leucine, and *l*-valine [22,23,24]. The fermentation method is well established for the amino acids *l*-threonine [25] and *l*-tryptophan [26], but recombinant strains of *E. coli* are used. Compared to enzymatic processes, the production of *l*-phenylalanine [27] and *l*-cysteine [28] through *E. coli* fermentation offers a more cost-effective system to meet the growing market demand. Most of the amino acids produced by the fermentation process use either *C. glutamicum* or *E. coli*, but other bacterium, such as *Brevibacterium flavum* and *Methylobacterium* sp., can also be used in the production of *l*-arginine and *l*-serine, respectively [8]. Fermentation processes have several advantages compared to the other methods. Only the biologically active *l*-forms of amino acids are produced, thereby avoiding further purification steps. Moreover, maintenance costs are significantly lower compared to the extraction processes [29]. Consequently, fermentation is the most commonly adopted process for industrial-scale *l*-amino acid production because of its economic and environmental advantages [6]. 

## 3. Soybean Crops and Soybean Meal Production 

Global soybean production has doubled in the past 20 years, reaching 347 million tonnes in 2017/18 (USDA data—published in March 2018 INFO Source Newsletter), of which the majority (82%) was produced in the USA, Brazil, and Argentina. The global usage of soybean meal was 234 million tonnes in the same year, where China, the USA, and the European Union were foremost. Chicken-meat production absorbs a substantial proportions of soybean meal as in the order of 44% of soybean meal fed to animals in the USA, and 32% in Europe, was offered to broiler chickens [30]. Global chicken-meat production has been predicted to double by 2050 [31] and has been projected to increase from 82 million tonnes in 2005/07 to 181 million tonnes in 2050 [32]. If this projection is valid, there will be a 72% increase chicken-meat production from 105.6 million tonnes in 2020 to 181.3 million tonnes in 2050. This, perhaps conservative, projection forecasts that an additional 76 million tonnes of chicken meat will be required in three decades time to meet global demand. Soybean meal is not the only source of protein available for inclusion in broiler diets, but it is the dominant feedstuff in this respect. An additional 76 million tonnes of chicken meat may require about 55 million tonnes of whole soybeans or 43 million tonnes of soybean meal, which is approaching 20% of the current global production. Some 55 million tonnes of soybeans would demand about 18 million hectares of arable land based on a yield of 3 tonnes per hectare, which was the case in the USA in 2005 [33]. While these estimates are approximate, they illustrate the chicken-meat industry’s dependence on soybean meal, and escalating prices could challenge the industry’s sustainability. Consequently, the need to seek viable alternatives becomes increasingly evident. 

## 4. Reduced-Crude Protein Diets 

The subject of reduced-CP diets seems to have emerged quite recently, but this perception is not sound. Non-bound methionine, lysine, and threonine have been routinely included in broiler diets for decades [34,35] and the availability of these non-bound amino acids has already allowed meaningful reductions in dietary CP and soybean meal inclusion levels in broiler diets [36], although this development may not have been recognised. However, the increasing commercial availability of the remaining proteinogenic amino acids should allow more tangible reductions in dietary CP and, in turn, greater declines in soybean meal inclusion levels.

An example of the composition and nutrient specifications of conventional (222 g CP/kg diet) and reduced-CP (165 g/kg) maize-based broiler diets from a recently completed feeding study is provided as Table 1. Inclusions of a range of non-bound amino acids were increased from 7.23 to 38.49 g/kg in formulating the reduced-CP diet; whereas, soybean meal inclusions were reduced by 66% (113 versus 334 g/kg). Importantly, the growth performance of male broiler chickens offered these diets from 7 to 35 days post-hatch was comparable (as yet unpublished data). The reduced-CP diet supported significantly higher weight gains by 7.05% (2370 versus 2214 g/kg) and feed intakes by 8.51% (3481 versus 3208 g/kg). This example unequivocally demonstrates the potential synthetic and crystalline amino acids hold as alternatives to soybean meal in chicken-meat production and is supported by another recent study [37]. Nevertheless, CP reductions of this magnitude may compromise the efficiency of feed conversion with associated increases in fat deposition [38,39,40,41]. The genesis of compromised feed conversion ratios (FCRs) probably stems, at least partially, from an insufficiently accurate identification of essential and non-essential amino acid requirements, or ideal protein ratios, in the context of reduced-CP diets. In addition to the different kinetics of non-bound versus protein-bound amino acids, the digestive dynamics of starch, protein, and lipid need to be considered, the relevance of which will probably become more evident with the development of reduced-CP diets [42,43]. Typically, dietary starch:protein ratios will be expanded in reduced-CP diets and the approach of limiting these increases in wheat-based broiler diets has shown some promise [44]. Another consideration is that while starch typically increases with reductions in dietary CP, on the other hand, dietary lipid levels decrease. Moreover, elevated starch to lipid ratios have been shown to influence broiler performance [45]. This could be of relevance in low-energy diets, where a further fat reduction would not be feasible with least-cost formulations. Additionally, when starch to lipid ratios exceed a certain threshold, this may prompt increased fat deposition in birds.

The quest to develop reduced-CP diets successfully is a complex challenge. However, it does appear that glycine and serine, threonine, and the branched chain amino acids isoleucine, leucine, and valine are the most challenging amino acids. Thus, these amino acids receive more attention in this review for this reason. 

## 5. Amino Acids

Amino acid requirements for broiler chickens are complex because, while amino acids are the ‘building-blocks’ of protein, they are also involved in a multiplicity of functional roles that are not directly related to skeletal protein deposition and growth [46,47]. There are further complications as it has been suggested that there are several ‘protein- or amino acid-sparing’ mechanisms in broiler chickens offered reduced-CP diets both at the digestive and at the post-absorptive level [48]. Moreover, it seems improbable that the post-enteral availability of a non-bound amino acid would precisely equal that of its protein-bound counterpart. This is because their digestive dynamics are fundamentally different; non-bound amino acids do not undergo digestion and are directly available for absorption in the upper small intestine and appear in the portal circulation more rapidly than protein-bound amino acids [49]. Non-bound methionine and lysine inclusions in sorghum-based broiler diets have been shown to accelerate their digestion rate constants relative to protein-bound amino acids [50]. This may mean that non-bound amino acids are less likely to undergo catabolism in the gut mucosa as has been suggested [51]. Ideal protein ratios or amino acid recommendations must be revised as new and compelling experimental data become available [47] and this equally applies to the new and different context that the advent of reduced-CP diets would create. 

Notionally, non-bound amino acids are totally ‘digestible’, and it has been concluded that the digestibility and bioavailability of crystalline lysine HCl in poultry is 100% [52]. However, apparent digestibility coefficients for methionine (0.890) lysine (0.974), and threonine (0.700) in caecectomised birds offered crystalline amino acid blends as the only protein source have been reported [53]. The corresponding true digestibility coefficients were 0.950, 0.974, and 0.956, respectively. Additionally, studies in birds with labelled non-bound *d,l*- and *l*-methionine have demonstrated complete absorption along the small intestine [54]. In the practical formulation of broiler diets, there is the implicit assumption that the digestibility of non-bound amino acids is 100%. Nevertheless, intestinal uptakes of non-bound amino acids might be less than 100% in the context of reduced-CP diets.

Intestinal uptakes of nutrients, including amino acids, or their absorption from the gut lumen into enterocytes of the small intestinal gut mucosa is pivotally important to broiler performance [55]. Single amino acids are absorbed via an array of some 14 Na^+^-dependent and 7 Na^+^-independent transport systems [56]. The complexity of absorption of amino acids, in this case methionine, is vividly illustrated in a quite recent review [57]. However, the majority of amino acids from ‘intact’ protein are absorbed as di- and tripeptides rather than single amino acids [58]. Moreover, the likelihood is that di- and tripeptides are absorbed more rapidly and efficiently via the oligopeptide transporter peptide transporter 1 (PepT-1) than single amino acids [59,60]. PepT-1 expression has been detected in poultry [61,62], but the relative importance of PepT-1 in the intestinal uptakes of amino acids in broiler chickens needs further clarification. Nevertheless, it seems probable that intestinal uptakes of oligopeptides derived from intact proteins may be advantaged in comparison to non-bound amino acids by the oligopeptide transporter, PepT-1. 

The post-enteral availability of amino acids is ultimately determined by their metabolic fates in the gut mucosa, where they may be captured by either anabolic or catabolic pathways and denied entry into the portal circulation as a consequence. Absorbed amino acids may be incorporated into mucin and digestive enzymes or be required to maintain gut integrity. However, amino acids are also critical energy sources for the gut mucosa [63], and the requirements of the digestive tract may account for some 20% of the incoming dietary energy [64]. Either amino acids, particularly glutamate and glutamine, or glucose are catabolised in avian enterocytes to meet the energy demand of the gut [65]. Glutamine and glucose provide similar proportions of energy to the gut mucosa in rats, but energy is probably derived more efficiently from glucose than amino acids [66]. The proportion of dietary amino acids that are catabolised may be in the order of 20%. This estimate is based on pig data, where the net portal outflow of ammonia accounted for 18% of the total amino acid nitrogen in weaner pig diets [67]. 

### 5.1. Impact of Dietary CP Reductions on Apparent Amino Acid Digestibility Coefficients 

In the majority of reduced-CP feeding studies, apparent and standardised amino acid digestibility coefficients are not assessed. However, CP reductions in maize-based diets have been shown to influence jejunal and ileal digestibility coefficients. A CP reduction from 210 to 165 g/kg increased the average jejunal digestibility of 16 amino acids by 29.4% (0.594 versus 0.459) [39] and the corresponding increase in ileal digestibility was 6.18% (0.790 versus 0.744). The average jejunal digestibility of 16 amino acids increased by 9.38% (0.758 versus 0.693) following a dietary CP reduction from 200 to 156 g/kg, with an increase of 5.84% (0.797 versus 0.753) in the ileum [40]. However, the average ileal digestibility of 16 amino acids declined marginally by 1.65% (0.776 versus 0.789) pursuant to a CP reduction from 208 to 165 g/kg as opposed to a 7.95% (0.665 versus 0.616) increase in the jejunum [41]. Thus, the overall amino acid digestibility increased by 14.1% in the jejunum and more modestly by 3.41% in the ileum following a reduction in dietary CP from 206 to 162 g/kg across three studies. The more pronounced increases in the jejunum may be attributed to the increased inclusions of non-bound amino acids and their notional 100% digestibility and proximal sites of absorption. However, the variation in increases from 7.95% to 29.4% cannot be explained readily but may reflect lower secretions of endogenous amino acid following reductions in dietary CP. Nevertheless, one implication is that these variations in the digestibility may generate amino acid imbalances at sites of protein synthesis.

### 5.2. The Cost of Deamination 

Skeletal protein turnover is an ongoing dynamic process, and consequently, protein synthesis and degradation or anabolism and catabolism of amino acids is taking place continuously [68,69]. Nevertheless, dietary amino acid imbalances will result in the catabolism of surplus amino acids and could impose costs of deamination [70]. Additions of imbalanced amino acid mixtures to reduced-CP diets was investigated in Hill and Olsen [71], and these researchers concluded that the resultant depressions in weight gain stemmed from the deamination of relatively large quantities of amino acids. Deamination principally involves hepatic oxidative deamination of amino acids, which generates ammonia. Ammonia is noxious and must be detoxified, where glutamine synthetase is pivotal as it catalyses the condensation of ammonia plus glutamate into glutamine [72,73]. Glutamine synthetase has been detected and quantified in poultry [74,75] and the condensation reaction catalysed by glutamine synthetase is shown in the following equation [76], which requires energy inputs:

NH_4_^+^ + Glu + ATP + Mg^2+^ → Gln + ADP + Pi. (1)

Glutamine then enters the Krebs uric acid cycle, which generates uric acid, and ammonia-N is excreted as uric acid-N. Inputs of glycine into the urea acid cycle are required, and serine, and possibly threonine, may serve as glycine precursors [77]. Every mole of uric acid synthesised as the end-product of N metabolism requires one mole of glycine [78], and the loss of glycine incurs a loss of 12.5 ATP molecules [75]. In mammalian models, inadequate ammonia detoxification and increased circulating ammonia, or hyperammonaemia, leads to reduced protein synthesis [71]. However, some poultry studies [79,80,81] suggest that elevated plasma ammonia concentrations depress the growth performance of broiler chickens. Indeed, it may be deduced that elevating plasma ammonia levels quadratically depressed weight gain (r = 0.755; *p* < 0.001) and FCR (r = 0.576; *p* = 0.039) when data from the last two studies are considered collectively. 

### 5.3. Glycine and Serine

The glycine–serine interrelationship in poultry was reported in Baker et al. [82] and their combined dietary concentrations may be expressed as glycine equivalents, where:

glycine equivalents_(g/kg)_ = glycine_(g/kg)_ + [serine_(g/kg)_ × 0.7143], (2)

which takes into account the difference in molecular weights. Very considerable interest in glycine in the context of reduced-CP diets was generated by the publication of Dean and colleagues [83]. Indeed, David Baker [78] described this work as a breakthrough, where the inclusion of glycine, in addition to essential amino acids, to a 160 g CP/kg diet resulted in comparable growth performance to the 220 g/kg maize-soy positive control diet. It is possible that amino acid imbalances and consequent deamination of surplus amino acids in reduced-CP diets will amplify the need for dietary glycine and uric acid synthesis and dietary glycine, although, almost by definition, there may be more deamination in high-protein diets [78]. 

Glycine metabolism has been reviewed [84], where glycine was considered to be a functional and conditionally essential amino acid because of its insufficient *de novo* synthesis. Numerous evaluations of glycine equivalents in reduced-CP broiler diets have been reported, and the responses are variable [85]. It has been proposed that glycine equivalents in diets for broiler chickens up to 21 days post-hatch should range between 11 and 20 g/kg [84]. This broad recommendation was justified on the basis that requirements for glycine equivalents are not constant and vary considerably depending on the dietary concentrations of threonine, arginine, cysteine, and choline. The lower end of the range is approached when the need to metabolise cysteine from methionine is low and when dietary concentrations of precursors like threonine and choline are high. There is also the likelihood that the quantity of N excreted as uric acid is a determinant of the requirement for dietary glycine equivalents [86,87,88]. 

One study is very relevant as the ratio of NH_3_-N to [NH_3_-N plus uric acid-N] in excreta was determined [88]. The addition of glycine equivalents to reduced-CP diets at all levels (132, 147, and 163 g/kg) decreased the ratio, indicating that glycine equivalents were used in uric acid synthesis. Moreover, it may be deduced from this study that NH_3_-N to [NH_3_-N plus uric acid-N] ratios were negatively related to weight gain in a linear manner (r = −0.918; *p* < 0.0001) and positively related to FCR in a linear manner (r = 0.928; *p* < 0.0001). Responses in broilers to dietary inclusions of glycine equivalents reported in the literature are variable and it appears that the extent to which glycine (and serine) are required to enter the uric acid cycle to synthesise uric acid may be an important contributor to this variability. Additionally, the highly significant linear relationships between NH_3_-N to [NH_3_-N plus uric acid-N] ratios recorded in Hofmann et al. [88] support the relevance of the cost of deamination and ammonia detoxification. 

### 5.4. Threonine

Non-bound threonine became commercially available in the 1980s and early evaluations in broiler chicks were reported in Suzuk and Mitsuhashi [89] and Smith and Waldroup [90], and the role of threonine in poultry was subsequently reviewed [91]. Interestingly, significant correlations between free threonine concentrations in portal plasma taken from the anterior mesenteric vein with both weight gains and feed efficiency in broilers from 7 to 28 days post-hatch have been reported [92]. 

Free threonine concentrations in systemic plasma have been found to increase substantially pursuant to a dietary CP reduction [93]. In this report, the transition from 183 to 159 g CP/kg diet diets increased free threonine concentrations in systemic plasma by 87% (1635 versus 876 nmol/ml) in female broiler chickens at 42 days post-hatch. Moreover, an increase in free threonine systemic plasma concentrations of 66% (1027 versus 619 µmol/L) at 34 days post-hatch following a reduction in dietary CP from 210 to 165 g/kg has been reported [39]. Similarly, an increase of 116% in plasma threonine (1093 versus 505 µmol/L) was observed pursuant to a dietary CP reduction from 200 to 156 g/kg [40]. These unequivocal increases in threonine concentrations raise the possibility that threonine dehydrogenase activity was being downregulated by reductions in dietary CP. Hepatic threonine dehydrogenase activity may be influenced by dietary protein levels, or other amino acids, to a greater extent than by threonine per se [94]. Instructively, a reduction in dietary CP from 320 to 230 g/kg triggered a 48.3% reduction in hepatic threonine dehydrogenase activity in broiler chickens, where both diets contained 6.7 g/kg threonine [95]. The reasons for the downregulation of threonine dehydrogenase activity and elevated systemic threonine plasma concentrations are somewhat obscure. However, threonine is an abundant amino acid in ileal endogenous flows in broiler chickens [96], and one reason for high threonine levels may be to recycle threonine to the gut mucosa for the synthesis of endogenous proteins, particularly mucin. Threonine is the dominant amino acid in porcine mucin [97].

Another salient point is that systemic threonine plasma levels in broiler chickens are relatively higher than their dietary levels. It may be deduced from one study [98] that threonine represented a 4.1% share of dietary amino acids but a 17.9% share of free amino acids in systemic plasma in broilers offered a casein-based diet with 180 g CP/kg diet. Threonine represented a 4.42% share (8.43 of 190.6 g/kg) of the total analysed dietary amino acid concentrations but a 10.0% share (505 of 5046 µmol/L) of the total amino acids in systemic plasma in birds offered the 200 g CP/kg diet in one study [40]. However, in birds offered the 156 g CP/kg diet, threonine represented a 5.46% share (7.74 of 141.8 g/kg) in the diet but a more substantial 19.6% share (1093 of 5563 µmol/L) in systemic plasma. These outcomes suggest that a better understanding of the genesis and purpose for elevated free threonine concentrations in the systemic plasma of birds offered reduced-CP diets is certainly required. 

In theory, threonine may serve as a precursor of glycine when threonine is enzymatically converted to glycine and acetaldehyde by threonine aldolase [99,100] and there are also interconversions between serine and glycine in poultry [101]. As discussed, increases of 66% and 116% in free threonine plasma concentrations following reductions in dietary CP have been reported [39,40]. However, plasma concentrations of both glycine and serine declined following dietary CP reductions in the same two experiments. The decreases were 26.6% (916 versus 1242 µmol/L) in Kidd and Tillman [34] and 32.2% (916 versus 1242 µmol/L) in Chrystal et al. [40] when expressed as glycine equivalents. The opposing shifts in free plasma concentrations of threonine and glycine equivalents do not appear to indicate that threonine was being converted to glycine. This outcome is supported [84,102], where it was concluded that *de novo* synthesis of glycine from threonine may be limited and threonine is not readily degraded to glycine and does not act as a precursor of this amino acid on the basis of the plasma amino acid data. 

Alternatively, it is widely held that threonine is converted to glycine and because of this, additional threonine will erode responses to glycine. For example, the addition of 4 g/kg glycine to five diets with CP contents ranging from 160 to 240 g/kg improved FCR by an average of 2.95% (1.315 versus 1.355), but the simultaneous addition of 4 g/kg threonine compromised FCR by 5.02% (1.381 versus 1.315) in [103]. The most pronounced glycine response was in birds offered the 180 g CP/kg diet as there was a 7.96% (1.342 versus 1.458) improvement in FCR, but this was depressed by 6.04% (1.423 versus 1.342) by the simultaneous addition of threonine. Alternatively, the suggestion from another study [104] was that additional threonine was not an effective means to overcome a deficiency in glycine equivalents in reduced-CP diets. 

The individual additions of either 1.10 g/kg non-bound threonine or 4.33 g/kg non-bound glycine equivalents to a 165 g CP/kg diet had little impact on broiler performance in Chrystal et al. [41]. However, the combined inclusions of threonine and glycine equivalents displayed promise as they increased weight gain by 7.82% (2150 versus 1994 g/kg; *p* < 0.025) and decreased relative fat-pad weight by 12.5% (11.65 versus 13.31 g/kg; *p* < 0.01). This outcome was not anticipated and, clearly, additional research to elucidate the mechanisms by which the threonine–glycine–serine axis influences broiler performance is justified. Therefore, threonine poses two quite different challenges. The first is to develop a better understanding of the elevations in free threonine systemic plasma concentrations in response to dietary CP reductions. The second is to clarify the relationship between threonine, glycine, and serine and to determine the extent to which threonine is a glycine precursor in broiler chickens offered reduced-CP diets.

### 5.5. Branched-Chain Amino Acids: Isoleucine, Leucine, and Valine

The branched-chain amino acids (BCAAs) are interesting in that either isoleucine or valine may be the fourth limiting amino acid in broiler diets; whereas, leucine levels are usually considered to be adequate. Moreover, there are antagonistic interactions between BCAAs, which have been identified in broiler chickens, although the underlying mechanisms have yet to be completely clarified. BCAA antagonism was probably first identified in poultry by Mathieu and Scott [105] and was subsequently investigated by other researchers [106,107]. Competition amongst BCAAs for intestinal uptake may be a contributing factor to their antagonism. Leucine inhibited the absorption of isoleucine and valine and isoleucine was shown to retard the absorption of leucine and valine in male rats from their concentrations in the intestinal lumen and portal plasma [108]. However, it is considered unlikely that BCAA antagonisms are an issue in broilers offered practical diets [109], but this may be declared in the context of reduced-CP diets, where excess leucine could trigger the catabolism of isoleucine and valine. Apparent BCAA digestibility coefficients in four small intestinal segments and their digestion rate constants in birds offered conventional sorghum-based diets are shown in Table 2. Only tyrosine has a slower digestion rate constant than the BCAA in Liu et al. [50]. This reflects the hydrophobicity of BCAAs, and the digestion rates of non-bound isoleucine, leucine, and valine may be disproportionately more rapid than their protein-bound counterparts, which could trigger amino acid imbalances. 

Three reviews provide instructive insights into the complexities of both leucine *per se* and the BCAAs in general [110,111,112]. The notion that the requirements for amino acids that are typically present in more than adequate amounts in broiler diets may not be adequately identified has been advanced [113]. Arguably, leucine is the prime candidate in this context as leucine ratios relative to lysine in the order of 130:100 are quite common in practical broiler diets, which comfortably exceed the Texas A&M optimal ratio of 109:100 [47].

Leucine is required for skeletal muscle deposition, but when plasma and intracellular leucine levels exceed the minimum needed for protein synthesis, then the metabolic roles of this amino acid declare themselves [114]. For example, high dietary leucine levels have been shown to activate the target of rapamycin signalling pathways (mTOR) in the skeletal muscle of neonatal chickens [115]. The activation of mTOR promotes protein synthesis in skeletal muscle and supresses protein catabolism [116]. In addition, leucine has been shown to reduce fat deposition in mice [117] and there is the contention that leucine plays a regulatory role in fat reduction [117]. Interestingly, there are interactions between leucine and glucose, and the glucose status may determine whether leucine is used in protein synthesis or for energy production [118,119]. Thus, the potential capacity of leucine to promote protein synthesis but supress fat deposition is of immediate relevance to the development of reduced-CP diets for broiler chickens. Thus, there is the implication that broiler chickens may benefit from higher dietary leucine inclusions, but it follows that this will necessitate higher inclusions of isoleucine and valine to counter antagonistic interactions between BCAAs. 

There are indications, albeit limited, that higher than standard BCAA concentrations may be advantageous in conventional and reduced-CP broiler diets. Concentrations of isoleucine (7.8 to 15.6 g/kg), leucine (14.7 to 26.2 g/kg), and valine (8.3 to 15.8 g/kg) were increased in 190 g CP/kg diet maize-soybean meal broiler diets containing 11.6 g/kg lysine [120]. This resulted in significant improvements of 20.0% in weight gain and 6.56% in feed efficiency in birds offered these diets from 7 to 21 days post-hatch. Young birds (6 to 20 days post-hatch) were offered 226 g/kg protein maize/soybean meal diets containing 14.7 g/kg lysine with three levels of BCAA concentrations [121]. Relative to lysine, this resulted in ratios of 79, 132, and 186 for leucine; 46, 77, and 109 for isoleucine; and 54, 91, and 128 for valine in contrast to the respective Texas A&M ratios of 109, 69, and 80. The high BCAA levels generated the greatest weigh gain; whereas, the intermediate BCAA levels generated the most efficient FCR and highest breast meat yields. Ratios in the order of 150 leucine, 87 isoleucine, and 103 valine appeared to be the most advantageous for FCR and breast meat yields; importantly, these levels comfortably exceed standard recommendations. 

Alternatively, increasing digestible leucine concentrations from an average of 14.6 g/kg to 16.9 and 20.6 g/kg in starter, grower, and finisher maize-soy diets with an average CP content of 196 g/kg offered to broilers from 1 to 34 days post-hatch did not influence growth performance in Zeitz et al. [122]. However, dietary concentrations of isoleucine and valine were kept constant in this study. In a subsequent study [123], isoleucine and valine were increased relative to the digestible leucine concentrations, which increased from an average of 12.6 g/kg to 16.4 and 19.6 g/kg in starter, grower, and finisher maize-soy diets, with an average CP content of 198 g/kg offered to broilers from 1 to 35 days post-hatch. Again, growth performance was not influenced by additional BCAA inclusions to diets that contained some peas, barley, and wheat in addition to maize and soybean meal. 

Increased fat deposition is an undesirable but consistent consequence in birds offered reduced-CP diets and this is associated with compromised FCR. The addition of 5.0 g/kg leucine to 200 and 180 g CP/kg diet maize-soybean meal diets significantly reduced the carcass fat composition by 16.4% (58.2 versus 69.6 g/bird) in broiler chickens at 42 days post-hatch. The average dietary leucine to lysine ratio was increased from 139 to 184 following the addition of 5.0 g/kg leucine [124]. Additionally, the dietary addition of 6.7 g/kg leucine was reported to reduce abdominal fat by 40.9% (69.8 versus 117.6 g/kg) [125]. It is noteworthy that increasing individual inclusions of leucine and valine have been shown to linearly decrease abdominal fat [81]. Increasing the leucine to lysine ratio from 98 to 171 linearly reduced the relative abdominal fat pad weights by 13.5% (35.8 versus 41.4 g/kg) and increasing the valine to lysine ratio from 53 to 107 generated a linear reduction of 18.8% (36.4 versus 44.8 g/kg) from 21 to 42 days post-hatch. The researchers concluded that the supplementation of leucine and valine can reduce abdominal fat deposition in birds fed reduced-CP diets during the grower phase. 

It is curious that the Texas A&M optimal amino acid ratio for grower pigs (20 to 50 kg liveweight) is 131 for leucine relative to lysine, but for grower broiler chickens (21 to 42 days post-hatch), the optimal ratio is much lower at 109. Given that leucine represents 6.83% of the whole-body protein in pigs and a nearly identical 6.92% in chicks [47], it is questionable as to why the leucine recommendation is noticeably lower in poultry. One possible implication is that leucine has more functional roles to perform in pigs than in poultry. Interestingly, the addition of 1.9 g/kg isoleucine, 1.0 g/kg leucine, and 3.4 g/kg valine to a 171 g CP/kg diet for weaner pigs generated huge responses of 64% in the average daily gain and 23% in the feed conversion efficiency in the 14-day post-weaning period [126]. These researchers concluded that the intestinal expression of amino acid transporters in weaner pigs is enhanced by BCAA supplementation of low-protein diets, which demonstrates the functionality of BCAAs in animal nutrition. 

### 5.6. Lysine

Non-bound lysine was commercially introduced in the 1970s, but it had been evaluated much earlier in broiler chickens [127]. In a series of landmark studies, Ted Batterham demonstrated that the daily duration of feed access negatively impacted on the efficiency with which lysine HCl is utilised in pigs [128,129,130,131]. That this phenomenon would extend to poultry was supported by data generated in [132] but not by data generated in Baker and Izquierdo [133]. Assuming Batterham’s findings are applicable to broiler chickens, the implication is that extending the daily hours of illumination would improve the utilisation of non-bound amino acids. This possibility was investigated, where it was found that providing birds with daily physical access to feed for intervals of 12, 16, and 20 h did not influence lysine HCl utilisation in broiler chickens [134]. The basis for this appears to lie in the capacity of birds to ‘crop-up’ via anticipatory feeding and retention of digesta in the crop [135]. Instructively, reducing feeding frequencies from 20 h to 16 and 12 h influenced the relative crop weights (*p* < 0.001), with increases from 3.8 g/kg to 5.5 and 7.7 g/kg [134]. There was a linear decline (r = 0.906; *p* *<* 0.001) in relative crop weights with increasing hours of feed access. Reducing feed access intervals also significantly increased the relative weights of empty gizzards and their contents. Therefore, it was concluded that the dichotomy between pigs and poultry in respect to lysine HCl utilisation appears related to crop and gizzard functionality and reverse peristalsis modulating the relative intestinal uptakes of non-bound versus protein-bound lysine. Thus, the implication is that the lighting regimen employed in grow-out facilities in practice is not likely to influence the utilisation of non-bound lysine specifically and, by extension, non-bound amino acids in general. 

### 5.7. Methionine 

The role of methionine in chicken-meat production has been reviewed [57,136]. Methionine is the first limiting amino acid for broilers offered maize-soybean meal diets and methionine was the first non-bound amino acid to be made commercially available. Methionine may be irreversibly converted to cysteine, and the sulphur-containing amino acids are required for protein accretion for both skeletal muscle deposition and feathering. Homocysteine is another sulphur-containing amino acid involved in the conversion of methionine to cysteine. Methionine is also a functional amino acid as methionine is involved in methyl donation and is a precursor of carnitine and glutathione to counter oxidative stress. In addition, methionine and cysteine positively influence the immune and inflammatory responses in poultry.

Non-bound methionine is produced by chemosynthesis and may be included in poultry diets as a powder (*d,l*-methionine or *l*-methionine) or as a liquid [*d,l*-2-hydroxy-4-(methyl) butanoic acid]. Recommended digestible methionine levels range from 4.0 to 5.1 g/kg for Ross 308 chickens, and as practical broiler diets often contain 2 to 3 g/kg non-bound methionine, it typically represents a tangible proportion of the total dietary methionine. Feathering is an obvious distinction between pigs and poultry and the likelihood is that fast-feathering strains require more cysteine, but not methionine, than slow-feathering broiler chickens [137]. This emphasises the importance of cysteine in relation to feathering, but excessive levels of cysteine are potentially toxic [138]. Moreover, the separate consideration of methionine and cysteine presents obstacles as cysteine concentrations may have profound effects on the magnitude of responses to methionine [78].

Graded amounts of either *d,l*-methionine, an equal blend of *d,l*-methionine and *l*-cysteine, or sulphur-containing amino acids derived from intact protein were added to a basal broiler diet with 208 g/kg protein in Huyghebaert and Pack [139]. Interestingly, non-bound methionine was more effective in improving weight gain and feed efficiency than non-bound methionine plus cysteine and, in turn, protein-bound methionine plus cysteine by substantial margins. Additionally, these researchers indicated that the requirements for a methionine plus cysteine increase with increases in the dietary CP contents, which implies the reverse may be the case with reduced-CP diets. Certainly, the addition of 1.76 g/kg non-bound methionine to a 165 g CP/kg diet broiler diet did not influence growth performance in Chrystal et al. [39]. However, enhanced utilisation of methionine in a reduced-CP diet (183 g CP/kg) compared to the standard protein diet (229 g/kg) offered to male broilers from 8 to 21 days post-hatch has been reported [140]. Maximum performance and protein deposition in the methionine dose–response studies were similar, but the maximal cumulative utilisation of methionine for deposition was 85% at 183 g CP/kg diet, but it was only 76% at 229 g CP/kg diet. Interestingly, maximal marginal methionine utilisations were achieved at 48% and 38% of the maximal deposition. At the point of maximal methionine deposition (95% of asymptotic response, requirement), the marginal utilisation of the last unit of added methionine was reduced to 21% and 19% in low and standard CP treatments, which demonstrated the law of diminishing returns. 

Interestingly, methionine is available as a dipeptide, which was developed for aquaculture. It may prove worthwhile to evaluate this methionine dipeptide in poultry because its intestinal uptake could be advantaged by the oligopeptide transporter, PepT-1, in comparison to monomeric synthetic methionine. 

## 6. Conclusions

Finally, our contention is that the development of diets for broiler chickens with CP reductions in the order of 50 g/kg may well be achieved provided sufficient research to both identify and address the limiting factors is completed. The development of “ideal protein ratios” that are relevant to reduced-CP, rather than conventional, diets will be required. Insofar as specific amino acids are concerned, we believe a better comprehension of the roles of threonine, glycine and serine, leucine, isoleucine, and valine in birds offered reduced-CP diets is necessary. However, they should not be considered in isolation from starch-protein digestive dynamics and perhaps, more particularly, the digestive dynamics of non-bound amino acids as opposed to protein-bound amino acids. Nonetheless, synthetic and crystalline amino acids will become viable alternatives to soybean meal in chicken-meat production if the objective is totally, or even partially, realised.

## Figures and Tables

**Table 1:** Composition and nutrient specifications of conventional (222 g Crude Protein, CP/kg diet) and reduced-CP (165 g CP/kg diet) maize-based experimental diets.

Composition Feed Ingredient (g/kg)	222 g CP/kg Diet	165 g CP/kg Diet	Nutrient Specifications Item (g/kg)	222 g CP/kg Diet	165 g CP/kg Diet
Maize	511	721	Crude protein	222	165
Canola seed	60	60	Starch	335	471
Soybean meal	334	113	Metabolizable energy (MJ/kg)	12.85	12.85
Soy oil	35	-	Calcium	8.25	8.25
*l*-lysine	1.60	8.12	Total phosphorus	7.20	6.84
*d,l*-methionine	2.67	4.53	Available phosphorus	4.13	4.13
*l-*threonine	1.18	4.10	Phytate phosphorus	2.47	2.02
*l*-tryptophan	-	0.79	Crude fat	85.1	54.1
*l-*valine	1.80	3.88	DEB (mEq/kg)	250	250
*l*-arginine	-	5.77	Digestible amino acids		
*l-*isoleucine	-	3.46	Lysine	11.50	11.50
*l*-leucine	-	1.41	Methionine	5.63	6.49
*l*-histidine	-	0.81	Cysteine	3.00	2.10
Glycine	0.32	3.57	Threonine	8.05	8.05
*l-*serine	0.01	3.84	Tryptophan	2.37	1.96
Sodium chloride	3.77	0.53	Isoleucine	8.19	7.94
Sodium bicarbonate	0.89	5.72	Leucine	16.29	12.54
Potassium carbonate	-	6.69	Arginine	12.96	12.42
Limestone	5.96	5.82	Valine	9.20	9.20
Dicalcium phosphate	21.2	24.4	Histidine	5.14	4.03
Choline chloride	0.90	0.90	Phenylalanine	9.63	5.58
Celite	20.0	20.0	Glutamic acid	33.07	19.33
Vitamin-mineral premix	2.0	2.0	Glycine	7.85	7.85
			Serine	9.32	9.32
Total non-bound amino acids	7.23	38.49	Glycine equivalents	14.51	14.51

**Table 2:** Apparent digestibility coefficients of branched-chain amino acids in four small intestinal segments and their digestion rate constants in birds offered sorghum-based diets (adapted from [44]).

Amino Acid	Apparent Digestibility Coefficient				Digestion Rate (× 10^−2^ Per Minute)
	Proximal Jejunum	Distal Jejunum	Proximal Ileum	Distal Ileum	
Isoleucine	0.363	0.568	0.726	0.774	2.05
Leucine	0.359	0.536	0.695	0.748	2.07
Valine	0.352	0.560	0.715	0.760	2.04

## References

[1] Mottet A., Tempio G. (2017). Global poultry production: Current state and future outlook and challenges. World’s Poult. Sci. J..

[2] Fiola N. (2008). Meeting the demand: An estimation of potential future greenhouse gas emissions from meat production. Ecol. Econ..

[3] Greenhalgh S., Chrystal P.V., Selle P.H., Liu S.Y. (2020). Reduced-crude protein diets in chicken-meat production: Justification for an imperative. World’s Poult. Sci. J..

[4] Kebreab E., Liedke A., Caro D., Deimling S., Binder M., Finkbeiner M. (2016). Environmental impact of using specialty feed ingredients in swine and poultry production: A life cycle assessment. J. Anim. Sci..

[5] Toride Y., Changchui H.E. (2004). Lysine and other amino acids for feed: Production and contribution to protein utilization in animal feeding. Protein Sources for the Animal Feed, Industry, Proceedings of FAO Animal, Production and Health, Bangkok, Thailand, 29 April–3 May 2002.

[6] Leuchtenberger W., Huthmacher K., Drauz K. (2005). Biotechnological production of amino acids and derivatives: Current status and prospects. Appl. Microbiol. Biotechnol..

[7] D’Este M., Alvarado-Morales M., Angelidaki I. (2018). Amino acids production focusing on fermentation technologies—A review. Biotechnol. Adv..

[8] Ikeda M. (2003). Amino acid production processes. Adv. Biochem. Eng. Biotechnol..

[9] Zhang J., Zhang S., Yang X., Qiu L., Gao B., Li R., Chen J. (2014). Reactive extraction of amino acids mixture in hydrolysate from cottonseed meal with di(2-ethylhexyl) phosphoric acid. J. Chem. Technol. Biotechnol..

[10] Breuer M., Ditrich K., Habicher T., Hauer B., Kesseler M., Stürmer R., Zelinski T. (2004). Industrial methods for the production of optically active intermediates. Angew. Chem. Int. Ed. Engl..

[11] Gröger H. (2003). Catalytic, Enantioselective Strecker, Reactions and Analogous. Syntheses. Chem. Rev..

[12] Fickler J., Heimbeck W., Hess V., Reimann I., Wiltafsky M., Zimmer U. (2016). AMINODat^®^ 5.0–Animal, Nutritionist’s Information.

[13] Chung T.K., Baker D.H. (1992). Utilization of methionine isomer and analogs by pig. Can J. Anim. Sci..

[14] European Food Safety Authority (EFSA) (2018). Safety and efficacy of hydroxy analogue of methionine and its calcium salt (ADRY+^®^) for all animal species. EFSA J..

[15] Pollegioni L., Servi S. (2003). Unnatural, Amino Acids, Methods and Protocols.

[16] Woltinger J., Karau A., Leuchtenberger W., Drauz K. (2005). Membrane reactors at Degussa. Adv. Biochem. Engin/Biotechnol..

[17] Zhao G., Gong G., Wang P., Wang L., Liu H., Zheng Z. (2014). Enzymatic synthesis of L-aspartic acid by Escherichia coli cultured with a cost-effective corn plasm medium. Ann. Microbiol..

[18] Sheldon R.A., Sanders J.P.M. (2015). Comparison of the sustainability metrics of the petrochemical and biomass-based routes to methionine. Catal. Today.

[19] Binder W., Friedrich H., Lotter H., Tanner H., Holldorf H., Leuchtenberger W. (1995). Tierfuttersupplement auf der Basiseiner Aminosäure und Verfahren zu dessen Herstellung. Patent.

[20] Rodehutscord M., Borchert F., Gregus Z., Pack M., Pfeffer E. (2000). Availability and utilization of free lysine in rainbow trout (Oncorhynchus mykiss): 2. Comparison of L-lysine HCl and L-lysine sulphate. Aquaculture.

[21] Jackson M. (2001). A closer look at lysine sources: L-lysine sulfate plus fermentation co-products. Feed Int..

[22] Blombach B., Schreiner M.E., Bartek T., Oldiges M., Eikmanns B.J. (2008). *Corynebacterium glutamicum* tailored for high-yield L-valine production. Appl. Microbiol. Biotechnol..

[23] Vogt M., Haas S., Klaffl S., Polen T., Eggeling L., van Ooyen J., Bott M. (2014). Pushing product formation to its limit: Metabolic engineering of *Corynebacterium glutamicum* for L-leucine overproduction. Metab. Eng..

[24] Yin L., Shi F., Hu X., Chen C., Wang X. (2013). Increasing l-isoleucine production in *Corynebacterium glutamicum* by overexpressing global regulator Lrp and two-component export system BrnFE. J. Appl. Microbiol..

[25] Debabov V.G. (2003). The Threonine Story. Adv. Biochem. Engin/Biotechnol..

[26] Ikeda M., Katsumata R. (1999). Hyperproduction of Tryptophan by *Corynebacterium glutamicum* with the Modified, Pentose Phosphate, Pathway. Appl. Environ. Microbiol..

[27] Cordwell S.J. (1999). Microbial genomes and “missing” enzymes: Redefining biochemical pathways. Arch. Microbiol..

[28] Liu H., Fang G., Wu H., Li Z., Ye Q. (2018). L-Cysteine, Production in Escherichia coli Based on Rational, Metabolic Engineering and Modular, Strategy. Biotechnol. J..

[29] Sugimoto M. (2010). Amino, Acids, Production, Processes.

[30] Dei H.K., Krezhova D. (2011). Soybean as a feed ingredient for livestock and poultry. Recent, Trends for Enhancing the Diversity and Quality of Soybean, Products.

[31] Kleyn R. (2019). Practical views on global meat chicken nutrition. Proc. Aust. Poult. Sci. Symp..

[32] Alexandratos N., Bruinsma J. (2012). World, Agriculture towards 2030/2050.

[33] Egli D.B. (2008). Comparison of corn and soybean yields in the United, States: Historical trends and future prospects. Agron. J..

[34] Kidd M.T., Tillman P.B. (2012). Feed additive mythbusters: How should we feed synthetic amino acids?. Proc. Aust. Poult. Sci. Symp..

[35] Kidd M.T., Tillman P.B., Waldroup P.W., Holder W. (2013). Feed-grade amino acid use in the United, States: The synergetic inclusion history with linear programming. J. Appl. Poult. Res..

[36] Pesti G.M. (2009). Impact of dietary amino acid and crude protein levels in broiler feeds on biological performance. J. Appl. Poult. Res..

[37] Lemme A., Hiller P., Klahsen M., Taube V., Stegemann J., Simon I. (2019). Reduction of dietary protein in broiler diets not only reduces n-emissions but is also accompanied by several further benefits. J. Appl. Poult. Res..

[38] Belloir P., Méda B., Lambert W., Corrent E., Juin H., Lessire M., Tesseraud S. (2017). Reducing the CP content in broiler feeds: Impact on animal performance, meat quality and nitrogen utilization. Animal.

[39] Chrystal P.V., Moss A.F., Khoddami A., Naranjo V.D., Selle P.H., Liu S.Y. (2020). Impacts of reduced-crude protein diets on key parameters in male broiler chickens offered maize-based diets. Poult. Sci..

[40] Chrystal P.V., Moss A.F., Khoddami A., Naranjo V.D., Selle P.H., Liu S.Y. (2020). Effects of reduced crude protein levels, dietary electrolyte balance and energy density on the performance of broiler chickens offered maize-based diets with evaluations of starch, protein and amino acid metabolism. Poult. Sci..

[41] Chrystal P.V., Moss A.F., Yin D., Khoddami A., Naranjo V.D., Selle P.H., Liu S.Y. (2020). Glycine equivalent and threonine inclusions in reduced-crude protein, maize-based diets impact on growth performance, fat deposition starch-protein digestive dynamics and amino acid metabolism in broiler chickens. Anim. Feed Sci. Technol..

[42] Liu S.Y., Selle P.H. (2017). Starch and protein digestive dynamics in low-protein diets supplemented with crystalline amino acids. Anim. Prod. Sci..

[43] Selle P.H., Liu S.Y. (2019). The relevance of starch and protein digestive dynamics in poultry. J. Appl. Poult. Res..

[44] Greenhalgh S., McInerney B.V., McQuade L.R., Chrystal P.V., Khoddami A., Zhuang M.A.M., Liu S.Y., Selle P.H. (2020). Capping dietary starch: Protein ratios in moderately reduced crude protein, wheat-based diets showed promise but further reductions generated inferior growth performance in broiler chickens from 7 to 35 days post-hatch. Anim. Nutr..

[45] Liu S.Y., Naranjo V.D., Chrystal P.V., Buyse J., Selle P.H. (2019). Box-Behnken optimisation of growth performance, plasma metabolites and carcass traits as influenced by dietary energy, amino acid and starch to lipid ratios in broiler chickens. PLoS ONE.

[46] Wu G. (2013). Functional amino acids in nutrition and health. Amino Acids.

[47] Wu G. (2014). Dietary requirements of synthesizable amino acids by animals: A paradigm shift in protein nutrition. J. Anim. Sci. Biotechnol..

[48] Swennen Q., Laroye C., Janssens G., Verbeke K., Decuypere E., Buyse J. (2007). Rate of metabolic decarboxylation of leucine as assessed by a L[1-^13^C_1_]leucine breath test combined with indirect calorimetry of broiler chickens fed isocaloric diets with different protein:fat ratio. J. Anim. Physiol. Anim. Nutr..

[49] Wu G. (2009). Amino acids: Metabolism, functions, and nutrition. Amino Acids.

[50] Liu S.Y., Selle P.H., Cowieson A.J. (2013). Protease supplementation of sorghum-based broiler diets enhances amino acid digestibility coefficients in four small intestinal sites and accelerates their rates of digestion. Anim. Feed Sci. Technol..

[51] Truong H.H., Chrystal P.V., Moss A.F., Selle P.H., Liu S.Y. (2017). Rapid protein disappearance rates along the small intestine advantage poultry performance and influence the post-enteral availability of amino acids. Br. J. Nutr..

[52] Izquierdo O.A., Parsons C.M., Baker D.H. (1998). Bioavailability of lysine in l-lysine HCl. J. Anim. Sci..

[53] Chung T.K., Baker D.H. (1992). Apparent and true amino acid digestibility of a crystalline amino acid mixture and of casein: Comparison of values obtained with ileal-cannulated pigs and cecectomized cockerels. J. Anim. Sci..

[54] Esteve-Garcia E., Austic R.E. (1993). Intestinal absorption and renal excretion of dietary methionine sources by the growing chicken. J. Nutr. Biochem..

[55] Croom W.J., Brake J., Coles B.A., Havenstein G.B., Christensen V.L., McBride B.W., Peebles E.D., Taylor I.L. (1999). Is intestinal absorption capacity rate-limiting for performance in poultry?. J. Appl. Poult. Res..

[56] Hyde R., Taylor P.M., Hundal H.S. (2003). Amino acid transporters: Roles in amino acid sensing and signalling in animal cells. Biochem. J..

[57] Mastrototaro L., Sponder G., Saremi B., Aschenbach J.R. (2016). Gastrointestinal methionine shuttle: Priority handling of precious goods. Iubmb Life.

[58] Matthews D.M. (1983). Intestinal absorption of peptides. Biochem. Soc. Trans..

[59] Daniel H. (2004). Molecular and integrative physiology of intestinal peptide transport. Annu. Rev. Physiol..

[60] Gilbert E.R., Wong E.A., Webb K.E. (2008). Peptide absorption and utilization: Implications for animal nutrition and health. J. Anim. Sci..

[61] Zwarycz B., Wong E.A. (2013). Expression of the peptide transporters PepT1, PepT2, and PHT1 in the embryonic and posthatch chick. Poult. Sci..

[62] Miska K.B., Fetterer R.H. (2019). Expression of amino acid and sugar transporters, aminopeptidase, and the di- and tri-peptide transporter PepT1; differences between modern fast growing broilers and broilers not selected for rapid growth. Poult. Sci..

[63] Reeds P.J., Burrin D.G., Stoll B., van Goudoever J.B., Vevey/Karger A.G. (2000). Role of the gut in the amino acid economy of the host. Nestle Nutr Workshop Ser Clin Perform Programme.

[64] Cant J.P., McBride B.W., Croom W.J. (1996). The regulation of intestinal metabolism and its impact on whole animal energetics. J. Anim. Sci..

[65] Watford M., Lund P., Krebs K.A. (1979). Isolation and metabolic characteristics of rat and chicken enterocytes. Biochem. J..

[66] Fleming S.E., Zambell K.L., Fitch M.D. (1997). Glucose and glutamine provide similar proportions of energy to mucosal cells of rat small intestine. Am. J. Physiol. Gastrointest. Liver Physiol..

[67] Stoll B., Henry J., Reeds P.J., Yu H., Jahoor F., Burrin D.G. (1998). Catabolism dominates the first-pass intestinal metabolism of dietary essential amino acids in milk protein-fed piglets. J. Nutr..

[68] Klasing K.C., Calvert C.C., Jarrell V.L. (1987). Growth characteristics, protein synthesis and protein degradation in muscles from fast and slow growing chickens. Poult. Sci..

[69] Sklan D., Noy Y. (2004). Catabolism and deposition of amino acids in growing chicks: Effect of dietary supply. Poult. Sci..

[70] Selle P.H., Chrystal P.V., Liu S.Y. (2020). The cost of deamination in reduced-crude protein broiler diets. Proc. Aust. Poult. Sci. Symp..

[71] Hill D.C., Olsen E.M. (1963). Effect of the addition of imbalanced amino acid mixtures to a low protein diet, on weight gains and plasma amino acids of chicks. J. Nutr..

[72] Hakvoort T.B.M., He Y., Kulik W., Vermeulen J.L.M., Duijst S., Ruijter J.M., Runge J.H., Deutz N.E.P., Koehler E., Lamers W.H. (2017). Pivotal role of glutamine synthetase in ammonia detoxification. Hepatology.

[73] Stern R.A., Mozdziak P.E. (2019). Differential ammonia metabolism and toxicity between avian and mammalian species, and effect of ammonia on skeletal muscle: A comparative review. J. Anim. Physiol. Anim. Nutr..

[74] Horn G.W., Featherston W.R. (1972). Influence of level and source of nitrogen intake on liver glutamine synthetase activity in the chick. J. Nutr..

[75] Watford M., Wu G. (2005). Glutamine metabolism in uricotelic species: Variation in skeletal muscle glutamine synthetase, glutaminase, glutamine levels and rates of protein synthesis. Comp. Biochem. Physiol. Part B.

[76] Minet R., Villie F., Marcollet M., Meynial-Denis D., Cynober L. (1997). Measurement of glutamine synthetase activity in rat muscle by a colorimetric assay. Clin. Chim. Acta.

[77] Salway J.G. (2018). The Krebs uric acid cycle: A forgotten Krebs cycle. Trends Biochem. Sci..

[78] Baker D.H. (2009). Advances in protein-amino acid nutrition in poultry. Amino Acids.

[79] Namroud N.F., Shivazad M., Zaghari M. (2008). Effects of fortifying low crude protein diet with crystalline amino acids on performance, blood ammonia level, and excreta characteristics of broiler chicks. Poult. Sci..

[80] Ospina-Rojas I.C., Murakami A.E., Duarte C.R.A., Eyng C., Oliveira C.A.L., Janeiro V. (2014). Valine, isoleucine, arginine and glycine supplementation of low-protein diets for broiler chickens during the starter and grower phases. Br. Poult. Sci..

[81] Ospina-Rojas I.C., Murakami A.E., Duarte C.R.A., Nascimento G.R., Garcia E.R.M., Sakamoto M.I., Nunes R.V. (2017). Leucine and valine supplementation of low-protein diets for broiler chickens from 21 to 42 days of age. Poult. Sci..

[82] Baker D.H., Sugahara M., Scott H.M. (1968). The glycine-serine interrelationship in chick nutrition. Poult. Sci..

[83] Dean D.W., Bidner T.D., Southern L.L. (2006). Glycine supplementation to low protein, amino acid-supplemented diets supports optimal performance of broiler chicks. Poult. Sci..

[84] Wang W., Wu Z., Dai Z., Yang Y., Wang J., Wu G. (2013). Glycine metabolism in animals and humans: Implications for nutrition and health. Amino Acids.

[85] Siegert W., Rodehutscord M. Relevance of glycine in low protein broiler feeds. Proceedings of the 20th European Symposium in Poultry, Nutrition, World’s Poultry, Science Association.

[86] Siegert W., Rodehutscord M. Nonessential amino acids—The forgotten nutrients?. Proceedings of the XVth European Poultry Conference, World’s Poultry, Science Association.

[87] Siegert W., Ahmadi H., Helmbrecht A., Rodehutscord M. (2015). A quantitative study of the interactive effects of glycine and serine with threonine and choline on growth performance in broilers. Poult. Sci..

[88] Hofmann P., Siegert W., Kenéz Á., Naranjo V.D.D., Rodehutscord M. (2019). Very low crude protein and varying glycine concentrations in the diet affect growth performance, characteristics of nitrogen excretion, and the blood metabolome of broiler chickens. J. Nutr..

[89] Suzuki M., Mitsuhashi T. (1982). Effects of methionine and threonine in diets on the growth and plasma free amino acids in chicks. Bull. Natl. Inst. Anim. Ind..

[90] Smith N.K., Waldroup P. (1988). Investigations of threonine requirements of broiler chicks fed diets based on grain sorghum and soybean meal. Poult. Sci..

[91] Kidd M.T., Kerr B.J. (1996). L-Threonine for poultry: A review. J. Appl. Poult. Res..

[92] Selle P.H., Truong H.H., McQuade L.R., Moss A.F., Liu S.Y. (2016). Reducing agent and exogenous protease additions, individually and in combination, to wheat- and sorghum-based diets interactively influence parameters of nutrient utilisation and digestive dynamics in broiler chickens. Anim. Nutr..

[93] Fancher B.I., Jensen L.S. (1989). Dietary protein levels and essential amino acid content: Influence upon female broiler performance during the growing period. Poult. Sci..

[94] Davis A.T., Austic R.E. (1997). Dietary protein and amino acid levels alter threonine dehydrogenase activity in hepatic mitochondria of *Gallus domesticus*. J. Nutr..

[95] Yuan J.-H., Austic R.E. (2001). The effect of dietary protein level on threonine dehydrogenase activity in chickens. Poult. Sci..

[96] Ravindran V., Hendriks W.H. (2004). Endogenous amino acid flows at the terminal ileum of broilers, layers and adult roosters. Anim. Sci..

[97] Lien K.A., Sauer W.C., Fenton M. (1997). Mucin output in ileal digesta of pigs fed a protein-free diets. Zeitschrift Ernährungswissenschaft.

[98] Tasaki I., Ohno T. (1971). Effect of dietary protein level on plasma free amino acids in the chicken. J. Nutr..

[99] Baker D.H., Hill T.M., Kleiss A.J. (1972). Nutritional evidence concerning formation of glycine from threonine in the chick. J. Anim. Sci..

[100] Graber G., Baker D.H. (1973). The essential nature of glycine and proline for growing chicks. Poult. Sci..

[101] Suguhara M., Kandatsu M. (1976). Glycine and serine interconversion in the rooster. Agric. Biol. Chem..

[102] D’Mello J.P.F. (1973). Aspects of threonine and glycine metabolism in the chick (*Gallus domesticus*). Nutr. Metab..

[103] Waldroup P.W., Jiang Q., Fritts C.A. (2005). Effects of glycine and threonine supplementation on performance of broiler chicks fed diets low in crude protein. Int. J. Poult. Sci..

[104] Hilliar M., Huyen N., Girish C.K., Barekatain R., Wu S., Swick R.A. (2019). Supplementing glycine, serine, and threonine in low protein diets for meat type chickens. Poult. Sci..

[105] Mathieu D., Scott H.M. (1968). Growth depressing effect of excess leucine in relation to the amino acid composition of the diet. Poult. Sci..

[106] Smith T.K., Austic R.E. (1978). The branched-chain amino acid antagonism in chicks. J. Nutr..

[107] Calvert C.C., Klasing K.C., Austic R.E. (1982). Involvement of food intake and amino acid catabolism in the branched-chain amino acid antagonism in chicks. J. Nutr..

[108] Szmelcman S., Guggenheim K. (1966). Interference between leucine, isoleucine and valine during intestinal absorption. Biochem. J..

[109] Waldroup P.W., Kersey J.H., Fritts C.A. (2002). Influence of branched-chain amino acid balance in broiler diets. Int. J. Poult. Sci..

[110] Li F., Yin Y., Tan B., Kong X., Wu G. (2011). Leucine nutrition in animals and humans: mTOR signaling and beyond. Amino Acids.

[111] Zhang S., Zeng X., Ren M., Mao X., Qiao S. (2017). Novel metabolic and physiological functions of branched chain amino acids: A review. J. Anim. Sci. Biotechnol..

[112] Nie C., He T., Zhang W., Zhang G., Ma X. (2018). Branched chain amino acids: Beyond nutrition metabolism. Int. J. Mol. Sci..

[113] Waldroup P.W. Do crude protein levels really matter?. Proceedings of the 15th Annual ASAIM Southeast, Asian Feed, Technology and Nutrition, Workshop.

[114] Harper A.E., Miller R.H., Block K.P. (1984). Branched-chain amino acid metabolism. Annu. Rev. Nutr..

[115] Deng H., Zheng A., Liu G., Chang W., Zhang S., Cai H. (2014). Activation of mammalian target of rapamycin signaling in skeletal muscle of neonatal chicks: Effects of dietary leucine and age. Poult. Sci..

[116] Zhang Y., Guo K., LeBlanc R.E., Loh D., Schwartz G.J., Yu Y.H. (2007). Increasing dietary leucine intake reduces diet-induced obesity and improves glucose and cholesterol metabolism in mice via multimechanisms. Diabetes.

[117] Duan Y., Li F., Liu H., Li Y., Liu Y., Kong X., Zhang Y., Deng D., Tang Y., Feng Z. (2015). Nutritional and regulatory roles of leucine in muscle growth and fat reduction. Front. Biosci..

[118] Shao D., Villet O., Zhang Z., Choi S.W., Yan J., Ritterhoff J., Gu H., Djukovic D., Christodoulou D., Kolwicz S.C. (2018). Glucose promotes cell growth by suppressing branched-chain amino acid degradation. Nat. Commun..

[119] Yoon I., Nam M., Kim H.K., Moon H.-S., Kim S., Jang J., Song J.A., Jeong S.J., Kim S.B., Cho S. (2020). Glucose-dependent control of leucine metabolism by leucyl-tRNA synthetase. Science.

[120] Yamazaki M., Murakami H., Nakashima K., Abe H., Takemasa M. (2006). Effects of excess essential amino acids in low protein diets on abdominal fat deposition and nitrogen excretion of the broiler chicks. J. Poult. Sci..

[121] Chen X., Zhang Q., Applegate T.J. (2016). Impact of dietary branched chain amino acids concentration on broiler chicks during aflatoxicosis. Poult. Sci..

[122] Zeitz J.O., Käding S.-K., Niewald I.R., Machander V., de Paula Dorigam J.C., Eder K. (2019). Effects of leucine supplementation on muscle protein synthesis and degradation pathways in broilers at constant dietary concentrations of isoleucine and valine. Arch. Anim. Nutr..

[123] Zeitz J.O., Käding S.-K., Niewald I.R., Most E., de Paula Dorigam J.C., Eder K. (2019). The influence of dietary leucine above recommendations and fixed ratios to isoleucine and valine on muscle protein synthesis and degradation pathways in broilers. Poult. Sci..

[124] Erwan E., Alimon A.R., Sazili A.Q., Yaakub H., Karim R. (2011). Effects of levels of L-leucine supplementation with sub-optimal protein in the diet of grower-finisher broiler chickens on carcass composition and sensory characteristics. Asian Aust. J. Anim. Sci..

[125] Erwan E. (2018). Supplementation if caloric- and protein-restricted diets with *l*-leucine stimulates food intake and improves carcass characteristics in broiler chickens. Int. J. Poult. Sci..

[126] Zhang S., Qiao S., Ren M., Zeng X., Ma X., Wu Z., Thacker P., Wu G. (2013). Supplementation with branched-chain amino acids to a low-protein diet regulates intestinal expression of amino acid and peptide transporters in weanling pigs. Amino Acids.

[127] Edwards H.M., Norris L.C., Heuser G.F. (1956). Studies on the lysine requirement of chicks. Poult. Sci..

[128] Batterham E.S. (1974). Effect of frequency of feeding on utilization of free lysine by growing pigs. Br. J. Nutr..

[129] Batterham E.S., O’Neill G.H. (1978). Effect of frequency of feeding on response by growing pigs to supplements of free lysine. Br. J. Nutr..

[130] Batterham E.S., Murison R.D. (1981). Utilization of free lysine by growing pigs. Br. J. Nutr..

[131] Batterham E.S., Bayley H.S. (1989). Effect of frequency of feeding of diets containing free or protein-bound lysine on the oxidation of [14^C^]lysine or [14^C^]phenylalanine by growing pigs. Br. J. Nutr..

[132] Nonis M.K., Gous R.M. (2006). Utilisation of synthetic amino acids by broiler breeder hens. S. Afr. J. Anim. Sci..

[133] Baker D.H., Izquierdo M.S. (1985). Effect of meal frequency and spaced crystalline lysine ingestion on the utilization of dietary lysine by chickens. Nutr. Res..

[134] Yin D., Chrystal P.V., Moss A.F., Choy K.Y.E., Liu S.Y., Selle P.H. (2019). Extending daily feed access intervals does not influence lysine HCl utilisation but enhances amino acid digestibilities in boiler chickens. Poult. Sci..

[135] Classen H.L., Apajalahti J., Svihus B., Choct M. (2016). The role of the crop in poultry production. World’s Poult. Sci. J..

[136] Bunchasak C. (2009). Role of dietary methionine in poultry production. J. Poult. Sci..

[137] Kalinowski A., Moran E.T., Wyatt C. (2003). Methionine and cystine requirements of slow and fast-feathering male broilers from zero to three weeks of age. Poult. Sci..

[138] Dilger R.N., Baker D.H. (2008). Excess dietary L-cysteine causes lethal metabolic acidosis in chicks. J. Nutr..

[139] Huyghebaert G., Pack M. (1996). Effects of dietary protein content, addition of nonessential amino acids and dietary methionine to cysteine balance on responses to dietary sulphur-containing amino acids in broilers. Br. Poult. Sci..

[140] Fatufe A.A., Rodehutscord M. (2005). growth, body composition, and marginal efficiency of methionine utilization are affected by nonessential amino acid nitrogen supplementation in male broiler chicken. Poult. Sci..

